# Joint malaria surveys lead towards improved cross-border cooperation between Savannakhet province, Laos and Quang Tri province, Vietnam

**DOI:** 10.1186/1475-2875-11-262

**Published:** 2012-08-03

**Authors:** Tiengkham Pongvongsa, Hoang Ha, Le Thanh, Ron P Marchand, Daisuke Nonaka, Bumpei Tojo, Panom Phongmany, Kazuhiko Moji, Jun Kobayashi

**Affiliations:** 1 Savannakhet Provincial Malaria Station, Savannakhet, Laos; 2 Quang Tri Province Preventive Medicine Center, Quang Tri, Vietnam; 3 Medical Committee Netherlands-Vietnam, Nha Trang, Vietnam; 4 Department of Parasitology and International Health, Graduate School of Medicine, University of the Ryukyus, Okinawa, Japan; 5 Research Institute for Humanity and Nature, Kyoto, Japan; 6 Savannakhet Provincial Health Department, Savannakhet, Laos; 7 Graduate School of International Health Development, Nagasaki University, Nagasaki, Japan; 8 Department of International Medical Cooperation, National Center for Global Health and Medicine, Tokyo, Japan

**Keywords:** Anopheles dirus, Forest cover, Long-lasting insecticide-treated net, Indoor residual spraying, International border

## Abstract

**Background:**

In Savannakhet province, Laos and Quang Tri province, Vietnam, malaria is still an important health problem and most cases are found in the mountainous, forested border areas where ethnic minority groups live. The objectives of this study were to obtain a better joint understanding of the malaria situation along the border and, on the basis of that, improve malaria control methods through better cooperation between the two countries.

**Methods:**

Fourteen villages in Savannakhet and 22 villages in Quang Tri were randomly selected within 5 km from the border where a blood survey for microscopic diagnosis (n = 1256 and n = 1803, respectively), household interviews (n = 400, both sides) and vector surveys were conducted between August and October 2010. Satellite images were used to examine the forest density around the study villages.

**Results:**

Malaria prevalence was significantly higher in Laos (5.2%) than in Vietnam (1.8%) and many other differences were found over the short distance across the border. Bed net coverage was high (> 90%) in both Laos and Vietnam but, while in Laos more than 60% of the nets were long-lasting insecticide-treated, Vietnam used indoor residual spraying in this area and the nets were untreated. Anopheles mosquitoes were more abundant in Laos than in Vietnam, especially many *Anopheles dirus* were captured in indoor light traps while none were collected in Vietnam. The forest cover was higher around the Lao than the Vietnamese villages. After this study routine exchange of malaria surveillance data was institutionalized and for the first time indoor residual spraying was applied in some Lao villages.

**Conclusions:**

The abundance of indoor-collected *An. dirus* on the Laos side raises doubts about the effectiveness of a sole reliance on long-lasting insecticide-treated nets in this area. Next to strengthening the early detection, correct diagnosis and prompt, adequate treatment of malaria infections, it is recommended to test focal indoor residual spraying and the promotion of insect repellent use in the early evening as additional vector interventions. Conducting joint malaria surveys by staff of two countries proved to be effective in stimulating better collaboration and improve cross-border malaria control.

## Background

Malaria has been decreasing in many parts of Laos and Vietnam, but it remains particularly endemic in remote, forest and forest fringe areas [1-3], which often occur along the border [4]. Malaria is often more difficult to control in border areas due to the more heavily forested, mountainous and inaccessible terrain, and because of unknown population movements across the border. In addition, these areas are most inhabited by ethnic minorities [5,6] with limited formal education [7] and, therefore, less accessible for health education efforts. Furthermore, malaria control strategies and policies as well as the quality and management of the health care systems and conventions in data collection may differ across national borders, making cross-border collaboration difficult. One such area, with a clear trend towards greater infection and malaria morbidity near the international border, is on the border between Savannakhet province of Laos and Quang Tri province of Vietnam.

The malaria control strategy on the Lao side is based on long-lasting insecticide-treated nets (LLITN) for vector control and reducing human-vector contact [8,9]. However, in this particular area the strategy on the Vietnamese side relies mainly on indoor residual spraying [10,11]. There are also differences in practice on malaria diagnosis and treatment between the countries. Laos relies mostly on rapid diagnostic tests (RDT) while Vietnam uses microscopy as a rule. In both Vietnam and Laos the policy is that malaria patients do not need to pay for anti-malarial medicines when seeking care from the public health sector. However, there are differences in the access to public health facilities, the role of the private sector and auxiliary costs related to malaria treatment. In 2004, artemisinin-based combination therapy (ACT) using Coartem® (artemether + lumefantrine) was first introduced as a pilot intervention for the first-line treatment of uncomplicated falciparum malaria in three southern provinces of Laos. Since 2008, the use of this ACT has gradually scaled-up to cover the whole public health sector including village health volunteers in the country. Quinine and artesunate injectables are available for the treatment of severe malaria in district and provincial hospitals [8], while only chloroquine and quinine are available in the private sector. Since 2009, ACT has also been expanded to the private sector by the pilot Public Private Mix project in Laos. In Vietnam, artemisinin was used for the first time in 1989 in Binh Phuoc province after which various kinds of artemisinin derivatives have been used for malaria treatment. The National Malaria Control Programme first recommended the use of locally produced “CV8” which was a combination of dihydroartemisinin with piperaquine and primaquine in 2003. In 2007, the recommendation was changed to only dihydroartemisinin plus piperaquine (as ‘Artekin’ and some other local trade names) after which this ACT became the standard first- line treatment of *Plasmodium falciparum* infections [12]. Chloroquine plus primaquine remained stipulated for *Plasmodium vivax* infections.

As border malaria is one of the biggest obstacles for malaria control and elimination in the Greater Mekong sub-region (GMS) countries, coordination and collaboration among neighboring countries is very important [13]. Although cross border studies are in place in the GMS countries to monitor drug quality and drug resistance, few attempts have been made to assess socio-economic and demographic characteristics, preventive and treatment-seeking behaviour, and cross-border movements of people living in a border area against the background of different health systems and malaria control policies on both sides of that border.

Therefore, a cross border survey on malaria was jointly designed, organized and analysed among the responsible preventive health services of Savannakhet province, Laos, and Quang Tri province, Vietnam. The objectives of this study were to obtain a better joint understanding of the main factors affecting the malaria situation along the border and, on the basis of that, improve and harmonize the local malaria control methods through more effective cooperation between the preventive health staff of the two countries.

## Methods

### Study sites and population

**Figure 1:**
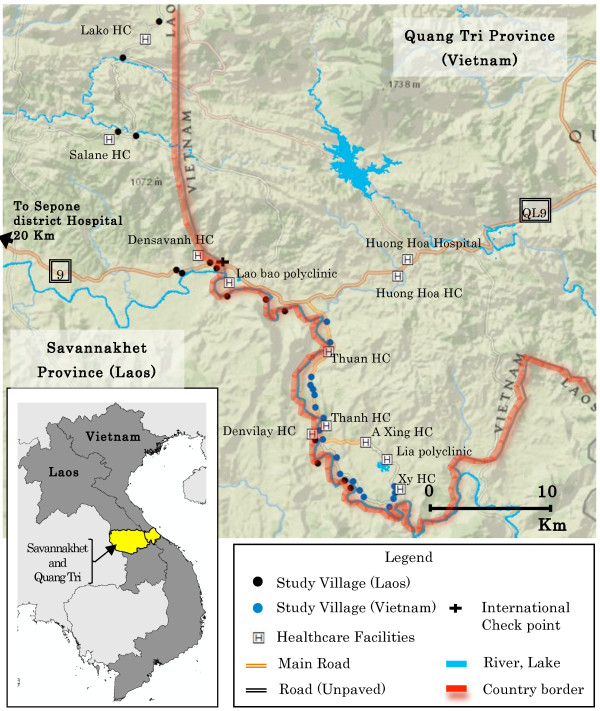
Location of selected villages, healthcare facilities, main road and river.

On the Lao side of the border, this study was conducted in the Sepon and Nong districts of Savannakhet province, which borders with Huong Hoa district in Quang Tri province, Vietnam (Figure 1). Savannakhet is located in the central part of Laos with a total population of 903,700 in 2011. In 2007, Savannakhet had a higher malaria incidence (5.2 per 1,000 person-years) than Quang Tri (2.6 per 1,000 person-years) [1]. In 2010, a total number of 10,334 positive malaria cases were recorded in Savannakhet province. Of these, 34.7% were reported from villages of the Sepon and Nong districts that lie along the river, which forms the border with Vietnam. Based on the meteorological reports for the Sepon district, the mean annual temperature, humidity and rainfall were 26.7°C, 78% and 1,382 mm, respectively. In these areas, many of the inhabitants are ethnic minorities (45% in Sepon; 95% in Nong). These groups, comprising the Tri and Makong peoples, have their own distinctive languages, and live in the remote mountainous and forested areas along the Laos-Vietnam border which are far from health care facilities and difficult to reach, especially during the rainy season [14]. 

To provide primary health care services at village level, at least two village health volunteers (VHVs) per village are trained on basic health care, including diagnosis and management of common diseases such as uncomplicated malaria, diarrhea and respiratory diseases. The trained VHVs are equipped with a kit for malaria diagnosis and treatment (RDT and Coartem®). They provide free malaria treatment services, assist in bed net impregnation/distribution, perform health education, collect and report malaria data as well as other important health events. They are regularly supervised by district and health centre staff. The RDT and ACT supply system was established by the Laos National Malaria Control Programme (NMCP), but sometimes it is difficult to supply on time because of several factors such as distance, transportation conditions and logistics management problems at the district and provincial levels. The VHVs in Laos are real volunteers and do not receive any regular monetary incentives from the government.

On the Vietnamese side of the border, the survey was conducted in Quang Tri province, which has a total population of 674,400. Over 9% of Quang Tri's inhabitants are ethnic minorities, predominantly Van-kieu and Paco people, who mostly live near the border with Laos [15]. In 2002–03, Quang Tri was among Vietnam's provinces with the highest malaria burdens with 4,178 cases [15]. In 2010, 850 positive malaria cases were recorded in the whole province, of which 723 (85.0%) were reported from villages in Huong Hoa district, which borders with the Sepon and Nong districts of Savannakhet, Laos. In 2010, based on the meteorological report of Huong Hoa district, the annual mean temperature, humidity and rainfall were 23.08°C, 87% and 2,938 mm, respectively. Similarly as in Laos, to provide primary health care services at village level, at least one or two village health workers (VHWs) are trained on basic health care for common diseases such as malaria, diarrhea, respiratory infection and first aid. In remote villages they are sometimes allowed to treat malaria patients with chloroquine and/or ACT but as a rule they have to refer patients to commune health centres. They primarily provide preventive health education, assist in the distribution of bed nets and facilitate other health programme activities in their community. They are supervised by district and commune health centre staff and regularly receive some incentives from several national health programmes.

In the main, southern part of the study area, the Lao and Vietnamese villages are separated by a river approximately 50 m wide, and surrounded by mountains and forests (Figure 1). Access to the villages is difficult from the Laos side but in Vietnam a road runs more or less parallel to the river. The inhabitants belong mostly to the same ethnic minority group, which is known as the ‘Tri’ in Laos and the ‘Van-kieu’ in Vietnam. They are allowed to freely cross the border by the governments of Laos and Vietnam. They have limited formal education but, besides their native language, often can speak the Vietnamese, much less the Lao national language. They frequently work in the forests for foraging, logging and swidden farming.

### Study design

This joint survey was carried out in Savannakhet (Laos) between August and September, 2010 while in Quang Tri (Vietnam) this was carried out in October of the same year. It was still the rainy season which usually shows the highest malaria transmission on both sides. In Laos, 14 villages with a total population of 4,576 individuals in 845 households were randomly selected from the Sepon and Nong districts by using a list of villages located within 5 km from the border river; 10 out of a total of 14 villages were in Sepon and four in Nong, each comprising a sample of 28–29 households. Within Huong Hoa district of Vietnam, three communes named Thuan, Thanh and Xy were chosen for this study in which 22 villages with a total population of 7,888 individuals in 1,427 households were randomly selected using the same sampling methods that were used for the Lao side, each comprising a sample of 18–19 households. From both sides of the border, 400 households from the study villages were selected for interviewing using a systematic random process.

Based on the questionnaire used in a previous malaria study performed along the Vietnam-Laos border [16], the standardized interview questionnaire was first developed in the English language by a joint group of Lao and Vietnamese local malaria experts. It was translated into the local Lao and Vietnamese languages, taking care to ensure consistency of meanings and definitions. This questionnaire was used to collect information from the selected household heads in the study villages on both sides of the border. It included questions related to socio-demographics, economic status, and knowledge, attitudes and practices regarding malaria prevention and control activities. The questionnaire was pre-tested on each side, and jointly adjusted where appropriate before conducting the survey. All the interviewers were trained in the use of the questionnaire and survey protocol by the team leaders of each side. The household head was a key respondent to the household questionnaire, but if he or she was absent any other household member aged over 18 years was asked to answer the questions. Before starting the interview, informed consent was clearly discussed and interviewee signatures or finger prints were obtained to indicate understanding and agreement. All respondents were interviewed at their homes, meeting face-to-face with the interviewer. For respondents who could not communicate with interviewers in the national language of each country, a trained translator was asked to translate the questions and answers in the native language.

The number of family members who were registered and targeted for blood examination was 2,435 in Laos and 5,701 in Vietnam. Other villagers who lived in the selected villages and were interested in knowing their malaria parasite status were also welcome to participate in the blood survey. A standard form based on the Active Case Detection survey forms of Laos and Vietnam was developed in English by a group of Lao and Vietnamese malaria staff and then translated into the two national languages. An informed consent form was prepared and discussed and participants’ signatures were obtained to indicate understanding and consent prior to blood sampling. For children, we obtained written informed consent from their guardians. Blood sampling was conducted door-to-door by a finger-prick for thick smear and RDT (Paracheck® Pf Rapid, Orchid Biomedical Laboratories, Goa, India). Rapid diagnostic testing was used only on the Lao side because the Vietnamese side did not have enough RDTs by the time of the survey. A thick blood smear sample was individually prepared and stained with 10% Giemsa for ten minutes in the field. The blood slides were first examined by local microscopists at the provincial malaria laboratories in each country. After this, all the slides from both countries were cross-checked by expert microscopists of the Quang Tri Preventive Medicine Center in Vietnam who had not been involved in the initial microscopic examination. All positive malaria cases were treated based on the national guidelines for malaria treatment of each country. Those on the Lao side who were detected to be malaria positive by rapid diagnostic test were immediately treated in the field. A list of those who were later found to be malaria positive by microscopic examination was sent to the local health centre for subsequent malaria treatment.

Due to geographical barriers and financial constraints, an entomological survey was conducted only in three villages on both sides. In Laos, the survey was implemented by a joint team of Lao and Vietnamese entomologists in Ban Cheng, Sepon district, where no blood survey was performed, and in Ban Denvilay and Ban Oi, Nong district, where the blood survey was only performed in Ban Denvilay. In Vietnam, the survey was implemented by Vietnamese entomologists in Ban 6 (Thuan commune), A Ho village (Thanh commune) and Xy Raman village (Xy commune). For this vector survey, an entomological survey form, based on the entomological survey forms of the NMCP of Laos and Vietnam, was developed in English language by a joint group Lao and Vietnamese experts, and translated into Lao and Vietnamese languages. Care was taken to ensure that consistent formats and definitions were used. The survey technique was based on the WHO guidelines [17]. Outdoor and indoor mosquito collection took place in 10 houses per village using three CDC light traps per house, one indoor and two outdoor (usually hung under the raised floor of houses on stilts), for five consecutive nights (from 6:00 p.m. to 7:00 a.m.) in each study village on both sides.

To better understand the relationship between forest density and the presence of malaria vector species in the study villages on both sides, Advanced Visible and Near Infrared Radiometer type 2 (AVNIR-2) satellite images taken from 10 February, 2007 were used in this study. For image classification, object-based classification as featured by the eCognition Developer Software was used [18,19]. Seven classes were defined including: 1) Water surface, 2) Semi-Closed forest, 3) Open forest, 4) Rangeland, 5) Closed forest, 6) Built-up areas (roads, houses etc.), and 7) Bare land. These seven land-cover features could be determined by visual interpretation of the color tone and texture pattern of false color composite satellite images.

### Data analysis

All collected data were checked, cleaned and entered into a computer by trained personnel using SPSS software version 17.5. The data were jointly analysed by Lao and Vietnamese teams. Descriptive statistics, the Pearson Chi-square and Mann–Whitney U tests were used to compare differences among household information and malaria related factors between Laos and Vietnam. A p value < 0.05 was considered to be statistically significant. The number of positive malaria cases, with slide positivity rate, malaria species, and malaria infection rate by age group and gender, were analysed and compared between Laos and Vietnam. The collected mosquitoes were morphologically identified in the field using the WHO standardized key for medically important Anophelines, and the national Identification Key for Anopheles in Vietnam 1987 prepared by the Institute of Malaria, Parasitology and Entomology, Hanoi [20]. Mosquito density was calculated as the mean number of mosquitos captured per light trap per night. The average amount of forest cover areas within 750 m and 1,500 m buffer zones from the centre of the study villages was calculated by using the classification results of the AVNIR-2 satellite images. For comparing the situation on both sides of the border, the closed forest and semi-closed forest areas (class 2 and 5) were grouped together (Table 1). 

**Table 1:** Total forest (ha) within 750 m and 1,500 m buffer zone from the centre point of each study village

	**Name of study village**	**Country**	**District name**	**Total forest (a)**	
				**Inside 750 m**	**Inside 1,500 m**
1	Alone	Laos	Sepon	3.2	105.6
2	Densavanh	Laos	Sepon	23.3	186.7
3	Pheung	Laos	Sepon	40.1	163.7
4	Kahanh	Laos	Sepon	31.8	189.1
5	Katoob Noy	Laos	Sepon	10.1	33.7
6	Katoob Gnai	Laos	Sepon	2.0	22.0
7	Mahard	Laos	Sepon	10.0	31.9
8	Kalad	Laos	Sepon	40.6	218.8
9	Sadoun	Laos	Sepon	28.3	90.5
10	Salene	Laos	Sepon	95.3	506.2
11	Vanglork	Laos	Sepon	112.9	487.8
12	Denvilay	Laos	Nong	16.1	119.8
13	Paliang Kao	Laos	Nong	38.9	166.5
14	Palobok	Laos	Nong	37.2	151.7
15	Palonam	Laos	Nong	52.1	195.4
16	Raman	Vietnam	Xy	51.4	262.9
17	Ta Nua	Vietnam	Xy	12.5	137.8
18	Troan O	Vietnam	Xy	5.5	61.3
19	Troan Thuong	Vietnam	Xy	0.5	14.6
20	Pa Lo Vac	Vietnam	Thanh	17.0	125.1
21	Sung	Vietnam	Thanh	70.7	252.3
22	To Nua	Vietnam	Thanh	26.0	186.0
23	Pa Lo O	Vietnam	Thanh	20.6	104.3
24	Thanh 4	Vietnam	Thanh	18.5	67.2
25	A Ho	Vietnam	Thanh	10.8	78.9
26	Thanh 10	Vietnam	Thanh	29.7	63.1
27	Thanh 9	Vietnam	Thanh	16.7	48.3
28	Thanh 8	Vietnam	Thanh	5.8	35.9
29	Ban 7	Vietnam	Thuan	3.6	34.7
30	Ban Zai	Vietnam	Thuan	5.1	55.3
31	Ban 6	Vietnam	Thuan	4.3	56.4
32	Ban 5	Vietnam	Thuan	4.2	51.3
33	Ban 2	Vietnam	Thuan	23.8	67.6
34	Ban 1 Cu	Vietnam	Thuan	15.3	52.7

a: Values indicate area (ha) in relatively dense forest classes (Class 2 and 5 of Figure. 1).

### Ethical clearance

This study was approved by the research ethical committees of the provincial health departments of Savannakhet, Laos and Quang Tri, Vietnam.

## Results

### Interview survey

Table 2 shows the results of interview survey on both sides of the border, 400 household heads completed interviews on socio-demographic and economic characteristics. In Laos, participants had a median age of 35 years, and the gender ratio was 61.2% male. In Vietnam, participants had a median age of 40 years, and the gender ratio was 50.5% male. The majority of participants were subsistence farmers engaged in swidden and/or paddy field farming. The median family size was the same on both sides with 6 persons per house. The majority of respondents on both sides were from the Tri/Van-kieu ethnic minority groups. During the survey, interviewers observed that most of the Lao and Vietnamese houses were built on stilts. Significant differences between the Lao and Vietnamese sides included materials of the house wall and possession of cattle: the walls were mostly made of wood (45.0%) or bamboo (49.0%) in the Lao houses, while wood (60.3%) was most commonly used in Vietnamese houses. On the Vietnamese side all respondents stated that they owned cattle while this applied to only 36.5% of the Lao respondents.

**Table 2:** Socio-demographic and economic characteristics of respondents

**Characteristics**	**Laos**		**Vietnam**		***P-value***
	**(n = 400)**		**(n = 400)**		
	**n**	**%**	**n**	**%**	
**Age group**					
< 25 years	68	17.0	62	15.5	*0.053* (a)
25– 35	145	36.3	135	33.8	
35– 45	93	23.3	87	21.7	
>45	94	23.4	116	29.0	
**Sex**					
Male	245	61.2	202	50.5	*0.002*
Female	155	38.8	198	49.5	
**Occupation**					
Farmer	359	89.8	398	99.5	*< 0.001*
Trader/business	20	5.0	1	0.3	
Others	21	5.2	1	0.2	
**Family size**					
≤ 2	11	02.7	07	1.7	*0.001*(a)
3–5	157	39.3	137	34.2	
6–8	184	46.0	183	45.8	
>8	48	12.0	73	18.3	
**Ethnicity**					
Tri/Van-kieu (b)	289	72.2	398	99.3	
Lao/Phouthai	53	14.3	0	0.0	
Paco	33	8.3	2	0.7	
Others	25	05.2	0	0.0	
**House type**					
Stilted house	364	91.0	378	94.5	*0.056*
Ground house	36	9.0	22	5.5	
**House wall**					
Wood	180	45.0	241	60.3	*0.001*
Brick	19	4.7	13	3.3	
Bamboo	196	49.0	146	36.4	
Others	5	1.3	0	0.0	
**Possession of cattle**					
Yes	146	36.5	400	100.0	
No	254	63.5	0	0.0	

a: Mann–Whitney *U* test.

b: Tri (Lao), Van-kieu (Vietnamese).

Table 3 shows the results of malaria related factors reported by respondents. Almost all households possessed at least one bed net in Vietnam (99.7%) and in Laos (92.2%) with an average of 2.3 and 2.6 persons/net, respectively. But, only 50% of Lao and 69% of Vietnamese respondents said they had enough nets for their needs. Most of the respondents in both sites reported that they slept under a bed net during the previous night. The time when family members usually retired to bed was between 8 p.m. and 12 p.m. on both sides. Seventeen percent of Lao and 14% of Vietnamese respondents reported to regularly spend nights out in the forest or field. Those who reported this were mostly male (83% in Laos and 88% in Vietnam). Thirty-nine percent of Lao and 30% of Vietnamese participants reported having experienced malaria in their families in the past year. Most of the respondents on both sides knew that mosquito bites are the main cause of malaria.

**Table 3:** Malaria related characteristics

**Characteristics**	**Laos**		**Vietnam**		***P-value***
	**(n = 400)**		**(n = 400)**		
	**n**	**%**	**n**	**%**	
**Possession of bed nets (all types)**					
No bed net	31	7.8	1	0.3	
1 – 2 nets	222	55.5	172	43.0	*0.001*
More than 2	147	36.8	227	56.7	
**Average of persons: 1 net**	2.6		2.3		
**Were net impregnated?**					
Yes	247	61.8	3	0.8	*< 0.001*
No	122	3.50	383	95.8	
Do not remember	0	0.0	13	3.2	
No bed net	31	7.7	1	0.2	
**Do you have enough bed nets?**					
Yes	199	49.8	275	69.0	*< 0.001*
No	170	42.5	124	31.0	
No bed net	31	7.7	1	0.2	
**Time family members usually go to bed (a)**					
Before 08 p.m.	189	47.3	87	21.7	*< 0.001*
08 – 11 p.m.	210	52.5	298	74.5	
After 11 p.m.	1	0.2	15	3.8	
**Slept under bed net last night**					
Yes	333	83.3	390	97.5	*< 0.001*
No	67	16.7	10	2.5	
**Used to stay overnight in the field**					
Yes	68	17.0	56	14.0	*0.31*
No	332	83.0	344	86.0	
**Slept under bed net when staying overnight in the field**	(n = 68)		(n = 142)		
Yes	20	29.4	50	35.2	*< 0.020*
No	47	69.1	76	53.5	
Do not remember	1	1.5	16	11.3	
**Distance of forest to house**					
< 100 m	278	69.5	152	38.0	
100–200 m	95	23.7	37	9.3	*<0.001*
>200 m	27	6.8	211	52.7	
**Any family member had malaria last 12 months?**					
Yes	157	39.3	122	30.5	
No	240	60.0	226	56.5	*< 0.001*
Do not remember	3	0.7	52	13.0	
**Treatment-seeking behaviour**					
Public sector of Vietnam	147	36.8	362	90.5	
Public sector of Laos	146	36.5	0	0.0	
Village Health Volunteer/Village Health Worker	22	5.5	32	8.0	
Self-medication	54	13.5	2	1.5	
Others	31	7.7	0	0.0	
**Malaria disease caused by**					
Flies	5	1.3	5	1.3	
Mosquito	289	72.3	328	82.0	
Earth/water/weather	7	1.7	9	2.3	
Go to forest	30	7.5	5	1.3	*< 0.001*
Others	38	9.5	4	1.1	
Do not know	31	7.7	49	12.0	
**No. of border crossings during the last 12 months**					
0	88	22.0	328	82.0	
1 – 50 times	171	42.8	72	18.0	*< 0.001*
50 – 100	98	24.5	0	0.0	
>100	43	10.7	0	0.0	
**Main reasons for crossing the border**	(n = 312)		(n = 72)	0.0	
Visit friends and relatives	76	24.3	23	32.0	
Seeking treatment or buying drugs	165	52.8	0	0.0	
Other business	71	22.7	49	68.0	
**Used bed net when staying overnight in another country**	(n = 88)		(n = 72)		
Always used	45	51.1	17	23.6	
Sometimes	10	11.4	16	22.2	*0.001*
Never used	33	37.5	39	54.2	

a: Ranged from 6 p.m. to 12 p.m. in Lao villages and from 8 p.m. to 12 p.m. in Vietnamese villages.

Major differences between Lao and Vietnamese sides included impregnation status of the nets, distance between the forest and the house, treatment-seeking behaviour, and number of time crossing the border. The majority (61.8%) of the nets in Laos were LLITNs, whereas there were no nets of this type in Vietnam and the nets in use by the people were rarely treated. It was reported that 94% of the Lao houses were located within 200 m from the forest edge whereas this was true for only 47% of the houses on the Vietnamese side. Most of the respondents (90.5%) in Vietnam relied on their own country's public health service compared to 36.5% in Laos. Another 36.8% of people living on the Lao side went to the public health facilities of Vietnam. The reliance on self-treatment and private pharmacies was 13.5% in Laos and less than 2% in Vietnam. The utilization of VHWs was quite low on both sides (5.5% in Laos and 8.0% in Vietnam). Eighty-eight percent of the Lao and 18% of the Vietnamese respondents reported to have crossed the border in the last year, and Lao respondents also overnighted more often in villages on the other side of the border. Fifty-one percent of Lao respondents who stayed overnight when they crossed the border slept under a bed net, whereas this was the case for only 23.6% of Vietnamese respondents. Among the Lao respondents, the most commonly reported reason for crossing the border was to seek treatment or to buy pharmaceutical drugs (52.8%), whereas the main reason among Vietnamese respondents was for business purposes (68.0%).

### Blood survey

The blood survey findings from both sides of the border are shown in Table 4. Blood samples for malaria parasite detection were collected from 1,256 persons in Laos and 1,803 persons in Vietnam (respectively 51.5% and 35.5% of the total population in the target households selected for this study). The slide positivity rate was significantly higher in Laos than in Vietnam (5.0% versus 1.8%; p = 0.001). On both sides, half of the infections were caused by *P. vivax* and half by *P. falciparum*. The malaria infection rate among children under 15 years was in Laos significantly higher than among adults, while in Vietnam the infections occurred equally among these age groups.

**Table 4:** Results of blood survey

**Indicators**	**Laos**		**Vietnam**		***P-value***
	**n**	**%**	**n**	**%**	
Total no. of population in the study villages	4,568		7,888		
No. of family members in the study households (target for blood survey)	2,435	53(a)	5,071	64(a)	
No. of blood examinations	1,256	51.5(b)	1,803	35.5(b)	*0.001*
No. malaria positive by microscopy	63	5.0	33	1.8	
**Malaria species**					
* P. falciparum*	30	47.6	18	54.5	*0.47(c)*
* P.vivax*	32	50.8	14	42.4	
Mix (*P. falciparum* + *P.vivax*)	1	1.6	1	3.0	
**Infection by age group**					
< 5 years (n: Laos =194; Vietnam = 251)	12	6.2	5	2.0	*0.022*
5 – 14 years (n: Laos = 349; Vietnam = 750)	33	9.5	12	1.6	*<0.001*
≥15 years (n: Laos = 713; Vietnam = 802)	18	2.5	16	2.0	*0.487*
**Infection by gender**					*0.52*
Male (n: Laos = 577; Vietnam = 816)	30	47.6	18	54.5	
Female (n: Laos = 679; Vietnam = 987)	33	52.4	15	45.5	

a: Compared to the total population of the study villages.

b: Compared to the total number of family members in the study households.

c: Between countries, disregarding the mixed cases.

### Entomology survey

In the vector survey, 490 mosquitoes belonging to fourteen Anopheles species were captured by CDC light traps in the Lao villages of which 51 (10.4%) were *Anopheles dirus* and 12 (2.5%) *Anopheles minimus*. Almost all (98%) of *An. dirus* and 42% of *An. minimus* were captured by the traps placed indoors. The density of *An. dirus* mosquitos was 2.56/trap/night and the density of *An. minimus* was 0.28/trap/night. In the Vietnamese study villages, 393 mosquitoes belonging to twelve different Anopheles species of which 21 (5.3%) were *An. minimus.* No *An. dirus* specimens were captured in the Vietnamese study villages and 71% of the *An. minimus* were captured in the outdoor traps. The density of *An. minimus* was 1.25/night/trap (Table 5).

**Table 5:** Results of entomological survey

**Variables**	**Laos (3 sites)**	**Vietnam (3 sites)**
Total number of mosquitoes captured	490	393
Anopheles species	14	12
No. of *Anopheles dirus*	51 (50 indoors)	0
No. of *Anopheles minimus*	12 (5 indoors)	21 (6 indoors)
Anopheles mosquito density (No./trap/night)	2.56 *(An. dirus)*	1.25 *(An. minimus)*

### Forest density surrounding study villages

**Figure 2:**
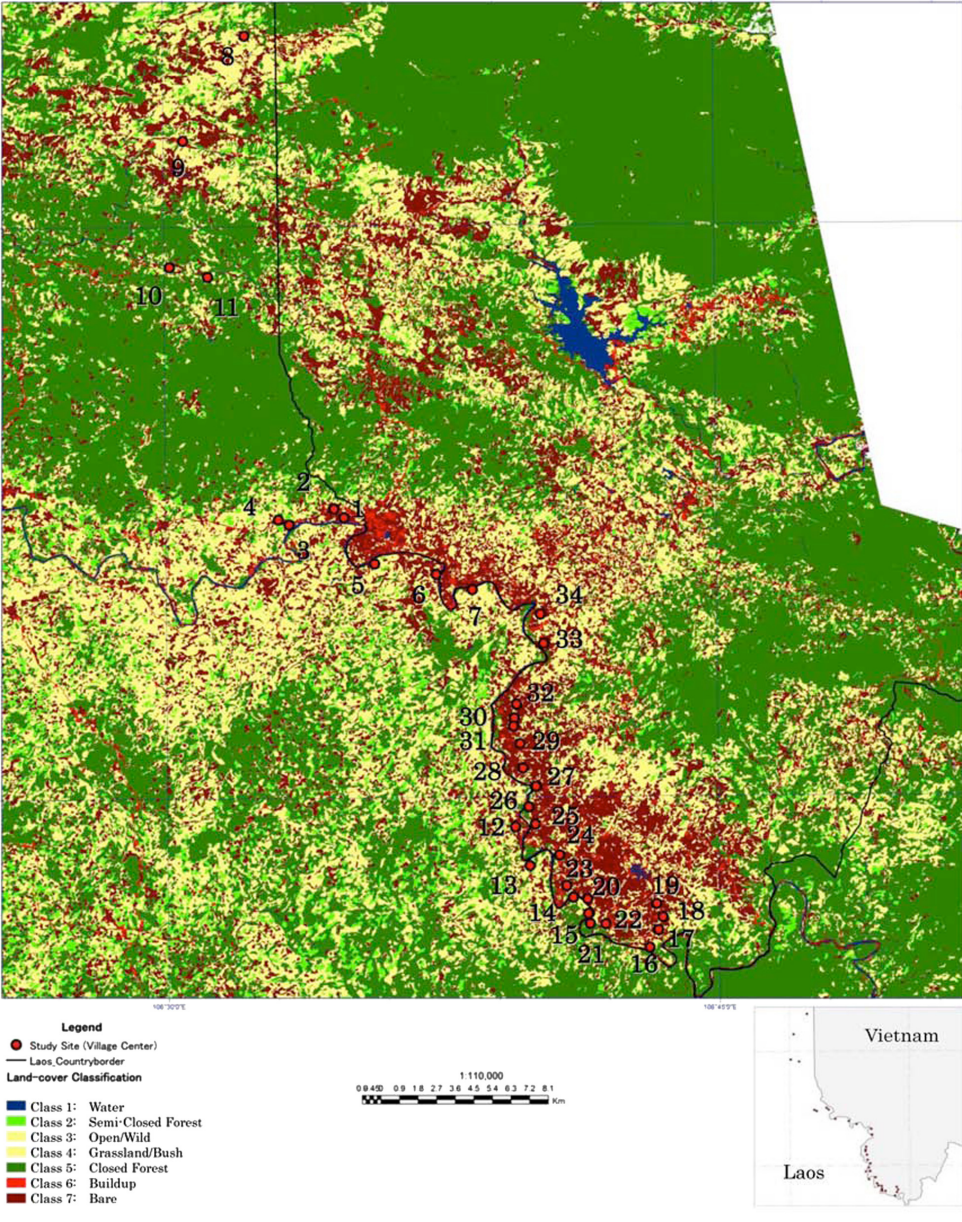
The distribution of land cover in the study site.

The distribution of forest cover is shown in Figure 2, which includes the location of the study villages on both sides of the border. As shown in Table 6, the average forest cover in Lao villages was approximately twice the average forest cover near the Vietnamese villages, both in the 750 m and 1.5 km buffer zones, confirming the data from the household survey about the forest edge at 100 and 200 metres distance from the houses.

**Table 6:** Average forest cover area inside 750 m and 1.5 km buffer zone of 15 villages on Laos side and 19 villages in Vietnam side

**Variables**	**Laos**	**Vietnam**
	**Median (IQR)(a)**	**Median (IQR)(a)**
Forest cover inside 750 m buffer zone	31.8 (13.1-40.35)	15.3 (5.3-22.2)
Forest cover inside 1,500 m buffer zone	163.7 (98.05-192.25)	63.1 (52–114.7)

Value indicates area (ha) in forest related classes (Class 2 and 5 from Figure 1) of the classification results of AVNIR2-images in 2007.

a: Median and interquartile range (25 %-75 %) of the forest related classes area in the whole 750 m and 1,500 m buffer zone area (176.6 ha and 706.5 ha, respectively).

### Improved cross-border collaboration

Two immediate and concrete outcomes of the intensified cooperation were: 1) agreement to have a regular exchange of malaria surveillance data in respect of: monthly malaria incidence data in the communes/villages on both side of the border, specifying the number of malaria cases among Lao persons detected by the Vietnamese health services; and 2) the joint carrying out of a round of Indoor Residual Spraying in the villages with the highest number of actual malaria cases at the Laos side in January 2011*.*

## Discussion

This study demonstrated some quite large differences between the Laos and Vietnamese respondents. At first sight this seemed surprising in view of the facts that 1) they belong to the same ethnic group (often with family relations across the border), 2) their villages are only a few kilometres from each other and 3) they easily and often cross the border. This applied to some socio-economic factors (e.g., the possession of cattle was much larger in Vietnam) and health related behavioural factors (e.g., health-seeking behaviour). On further reflection however, it is clear that an international border between two very different countries itself can create contrasts within the communities divided by it. The more easily accessible and more attractive health facilities on the Vietnamese side naturally caused the asymmetry in health-seeking behaviour. This was already known long before and one of the reasons for the need to improve cross-border collaboration in this area. The records of the Commune Health Centres in Vietnam have, for many years, shown a high proportion of Lao out-patients. It was clear from the Lao respondents that they preferred Vietnamese health service due to a combination of easier to reach and more affordable treatment. Other factors, like the perception about the quality of treatment and the language, are also likely to play a role. In comparison, more than half (68%) of the border crossing population on the Vietnamese side reported crossing the border to Laos for business purposes and none for seeking health care. Ironically, while borders are per definition designed to restrict people’s movements, they also create certain special livelihood opportunities (trading/smuggling) that stimulate people to cross it.

The environmental/biological differences over a short distance were in fact the more unexpected ones: higher malaria prevalence, different age distribution of infections, higher mosquito densities, different vector composition and higher forest cover on the Lao side. The aim of this study was to measure and, if possible, explain these differences in relation to the environmental and human factors, with a view to improve malaria control in the border area.

The blood survey showed that malaria prevalence was lower than expected on both sides, but it was significantly higher in Laos than in Vietnam. Both *P. falciparum* and *P. vivax* were found but the proportion of *P. vivax* to *P. falciparum* (equal) was greater than is usually found in the nationwide records in both countries. In Laos especially, *P. falciparum* is reported to account for 95% of all recorded malaria cases [21,22]. However, only provincial and district hospitals, and a few health centres, have microscopists who can perform species-specific diagnosis in Laos. In addition, as complications and severe disease resulting from *P. vivax* infection are rare and patients probably seek treatment less frequently, the passive case detection system is likely to underestimate the prevalence of *P. vivax* infection [23]. To improve case management at the community level the NMCP of Laos introduced between 2005 and 2008 a *P. falciparum* -specific malaria rapid diagnostic test that does not detect *P. vivax *[22], so that the number of *P. vivax* cases was underestimated even more since that time. However, in 2010, NMCP of Laos has revised the national strategy for malaria control and pre-elimination from 2011 and 2015. One of the objectives in this strategy is to improve access to early and accurate diagnosis for malaria by strengthening the public sector microscopy network and provide RDTs that can distinguish *P. falciparum* and *P. vivax* for the diagnosis at all “stratum 3 villages” (with high malaria incidence). Through the implementation of this strategy the determination of the *P. vivax* infection rate as well as species-specific treatment are likely to be improved in Laos [8].

Malaria infection rates in Laos were found to be higher in children under 15 years of age than in adults and the prevalence was highest in the age group from 5 to 14 years, whereas in the sample from Vietnam children and adults were equally likely to be infected. This suggests that there is a difference in acquired immunity status between the two populations as a result of a more intensive exposure to malaria infections in Laos. If this is true we would also expect a higher rate of asymptomatic malaria on the Laos side, which was not systematically checked to allow a comparison in this study.

Overnighting in the forest was only slightly more reported by respondents from Laos (17%) than from Vietnam (14%) and was on both sides mostly done by the males. However, in both countries the infection rates were not significantly different by sex. In combination with the high prevalence among children this suggests that at least on the Lao side most malaria is transmitted in the villages and not mainly incurred during work in the forest. A limitation of the study is however that we cannot know the proportion and infection status of people that were missed due to being away in the forest at the time of the survey. In Vietnam, it has been reported that regular sleeping in the forest increased the risk of malaria infection up till eight times and was more incurred by men than women [24]. However, this increased risk factor often only appeared after the transmission risk in the villages had been reduced, for instance by effective control of *An. minimus *[25].

With respect to malaria prevention measures, this study showed that the possession of bed nets by households was high on both sides, but that the coverage of insecticide-treated nets was much higher in Laos than in Vietnam. ITN also form the mainstay of prevention by the Vietnamese Malaria Control Program but when it is observed that too few people (less than 80–75%) actually sleep under a net in highly malarious area, or when this is considered a remote or otherwise problematic area, indoor residual spraying is applied. This border area is one of those areas where Vietnam continued to use IRS and where, while most people reported to have slept under a net, these were not insecticide treated. Not using IRS may be one of the reasons for the higher malaria prevalence on the Laos side, even though it has been reported that untreated bed nets also can have a significant protective effect on the risk of clinical malaria and malaria infection [24,26].

Another factor is the utilization rate of the available nets. During this survey no direct observations of bed net usage were made but the number of interviewed householders who reported that people had slept under the nets during the previous night was lower in Laos (83%) than in Vietnam (98%). Since 1999, the NMCP of Laos has distributed only the x-family size bed nets to the target villages with a target average coverage of 2.5 persons per net. This distribution ratio may not have provided enough bed nets for everyone in the family, especially in the poor ethnic minority villages where they could not buy an extra bed nets from the local market, and also where the sleeping behaviour is different from other groups due to local custom and culture. For instance, it has been noted from this area that the husband and wife do not regularly want to share the same bed net. This may explain the lower rate of people who reported to have slept under a bed net during the previous night at the Laos side.

However, more important is that the time of usually going to bed was reported to be between 8:00 and 11:00 pm on both sides. This still leaves some hours during which they are at risk of biting by Anopheles mosquitoes, especially by *An.dirus* which is often reported to start to bite early in the evening [25,27]. Therefore, even full utilization of bed nets may still be ineffective in reducing malaria transmitted by *An. dirus*.

The short entomological survey showed the presence of malaria vector mosquitoes on both sides of the border, but that their density was considerably higher in Laos than in Vietnam. Especially, the occurrence of a high density of *An. dirus* in the villages in an area at the Laos side where most malaria infections were found, while none were found during this survey in Vietnam, leaves no doubt about the role of this vector in explaining the difference in malaria prevalence. Less easy is it to explain the difference in the occurrence of *An. dirus* between villages that are near to each other just across the border, such as the Denvilay and Oi villages in Laos next to the A Ho and Xung villages in Vietnam. The indoor residual spraying that was applied at the Vietnamese side could be a factor, but this is not certain because the types of pyrethroid insecticides used in both IRS and LLITN are not known to strongly deter mosquitoes from entering houses. This study suggests that the presence of LLITN/ITN on the Lao side does not prevent *An. dirus* from entering houses and be captured by indoor light traps. This points to the urgent need to study the actual utilization of bed nets by people and the effectiveness of the used insecticides on reducing the exposure to mosquito bites, none of which were studied here. In general too little is known about the behaviour of *An. dirus,* as the most effective malaria vector in Southeast Asia [4,25,28-30] in response to different applications of insecticide. Its well-documented exophily [31-33] would make it less affected by any indoor insecticide use, certainly in comparison with *An. minimus*. The high density of *An. dirus* in indoor light traps found in this study is especially disturbing because of the sole reliance on LLITN for vector control in the Laos malaria control programme.

The geographic distribution of malaria prevalence obtained through the blood survey showed that the highest intensity of malaria transmission (on both sides of the border) occurred in the southern part of the study area. This was also the area that had the highest forest cover at the Laos side and is continuous with a relatively large, still uninhabited forest in Laos. This indicated a priority area for intensified control measures, especially at the Laos side.

The difference between the villages at the Laos and Vietnamese side of the border in respect of forest cover (by analysis of satellite pictures and the average distance of houses to the forest edge) found in this study fully matches with the strong association of *An. dirus* with the forest [25,34,35] and this may yet be the overriding factor for its presence in the Lao villages.

A limitation of this study was that the time at which the survey was conducted in the two countries differed by almost one month due to internal logistical factors of each country. However, according to the local meteorological data of each country, there was little seasonal variation in this border area during the period of study. Another limitation was that, although it was tried to recruit equal numbers of male and female respondents to participate in the interview, in Laos often the wife preferred her husband to answer when he was present (more men than women can speak the Lao or Vietnamese language). It is not known to what extent this has influenced the results of the interviews, nor can we assess whether the fact that all interviewers were male has influenced this.

Although it was not an objective of this study to assess the quality of the RDT diagnosis, a considerably proportion of false negatives was noted (13 out of the 35 persons microscopically positive for *P. falciparum* on the Laos side; the sensitivity was 63% and specificity was 99%). This implies that these microscopically positive people were not promptly treated during the survey if they had no symptoms either. It is, therefore, strongly recommended to study this separately in the future, preferably with the new RDTs that will be introduced. It is recommended to continue to strengthen the role and function of the VHVs, especially those at the Lao side, where they are more needed due to the scarcity of Health Zone Centres. The interview data showed that VHVs are under-utilized on both sides (Table 3). A prime necessity is that they are trusted by the local population and perceived as useful by the health sector, which remains difficult if they can not perform reliable diagnosis nor always have free anti-malarial medicines of the correct type.

This survey was the first cross-country survey jointly designed and conducted by staff from two provincial branches of the Malaria control services in Laos and Vietnam. Despite the limitations and obstacles encountered during the survey, this proved to be an effective initiative in cross border collaboration. It helped to focus the malaria staff of the two neighboring countries on a common evidence base in stead of each side talking from their own, often difficult to compare, data, ideas and prejudices. It led to more mutual appreciation of the particular constraints the other side had to face and set their minds to overcome or accept their differences and find a common solution to battle the malaria problem in their border area. It has led to a concrete intensification of the coordination (sharing malaria data) and even in an actual adjustment of the control methodology used on the Laos side (an IRS campaign in a hot spot along the border). Therefore we think this approach can be a good model to mediate effective cross-border collaboration also in other areas. In this case the intermediary role of an NGO (Medical Committee Netherlands-Vietnam) with experience of working in both countries was instrumental to facilitate the process while guarding the equivalence of the two partners in the joint project, each of which had their own strengths and weaknesses.

To improve the situation of malaria along this border area, an effective package of cross-border malaria control interventions needs to be developed and piloted on both sides. Based on the results of study it is recommended to conduct IRS in Laos not only in response to outbreaks but also allow more routine use of IRS in some villages in remote areas with continuous intense malaria transmission. Secondly, in addition to free distribution of LLITN, the use of insect repellents should be tested and popularized to reduce the biting risk in the hours that people are not yet under the nets. Thirdly, to improve bed net utilization both x-family and single sizes should be provided. Self-evidently this should all go hand in hand with a continued strengthening of routine anti-malaria activities such as the full provision of free malaria diagnosis and effective treatment in the public sector and the development of materials for information, education and communication (IEC) for malaria prevention and control that can be understood in Lao, Vietnamese and (for local radio and TV) also in the ethnic minority languages.

## Conclusions

The presence of *An. dirus*, which is known to be a highly efficient malaria vector, is likely to cause the higher prevalence of malaria in Laos compared with Vietnam. Forest coverage on the Lao side was higher than on the Vietnamese side of the border, and this is likely to favor the abundance of *An. dirus* on the Laos side. The finding of high densities of *An. dirus* in indoor light traps in Laos urgently requires further studies about the effectiveness of LLITN/ITN in this area. Local vector and environmental factors need to be taken into account to make malaria control along the border more effective and under special circumstances deviations from countrywide policies need to be considered. Based on the results of this study it was especially recommended to try out the focal use of IRS on the Laos side, to initiate the testing and promotion of insect repellents and to forcefully strengthen the access of all people to reliable and accurate diagnosis and free provision of treatment with ACT.

This type of joint, basic malariological studies by local staff responsible for malaria control on both sides of the border proved to be very effective in encouraging better collaboration in border malaria control.

## Competing interests

The authors declare that they have no competing interests.

## Authors’ contributions

TP, HH, LT and RPM were responsible for the entire process. DN supported data analysis and helped to review the manuscript. PP contributed to the development of the study design. KM and JK and TB contributed to the development of the study design, analyse and write the results of forest cover by using the satellite images and review the manuscript. All authors read and approved the final manuscript.
